# Orthopaedic Trauma Care in Gaza: Malaysian Emergency Medical Teams’ Experience and Challenges

**DOI:** 10.5704/MOJ.2603.024

**Published:** 2026-03

**Authors:** ZF Zairul-Nizam, KAH Abdul-Ghani, M Masniza

**Affiliations:** 1 Department of Orthopaedics, International Medical University School of Medicine, Seremban, Malaysia; 2 Department of Orthopaedics, Tengku Ampuan Rahimah Hospital, Klang, Malaysia; 3 Health Coordination Unit, MERCY Malaysia, Kuala Lumpur, Malaysia

Dear editor,

Since the Israeli onslaught on the occupied lands of Palestine—particularly the Gaza Strip beginning in October 2023—the world has witnessed catastrophic loss of life and widespread destruction. Gaza’s healthcare system has been among the most devastated sectors. By October 2025, over 90% of healthcare facilities in Gaza were destroyed or unable to deliver effective services^1^. The remaining healthcare professionals continue providing essential care with extremely limited resources, further strained by severe restrictions on vital medical equipment and medications.

In 2024, MERCY Malaysia Emergency Medical Team Type 1 Fixed (MMEMT), under the auspices of the World Health Organization (WHO), was permitted entry into the Gaza Strip via the Rafah Crossing^2^. From February to June 2024, MMEMT deployed four Specialist Specialized Care Teams (SCT) based at the Kuwaiti Specialist Hospital in Rafah. These teams primarily supported surgical disciplines, including anaesthesiology, general surgery, obstetrics, emergency medicine, and orthopaedics.

**Fig. 1:**
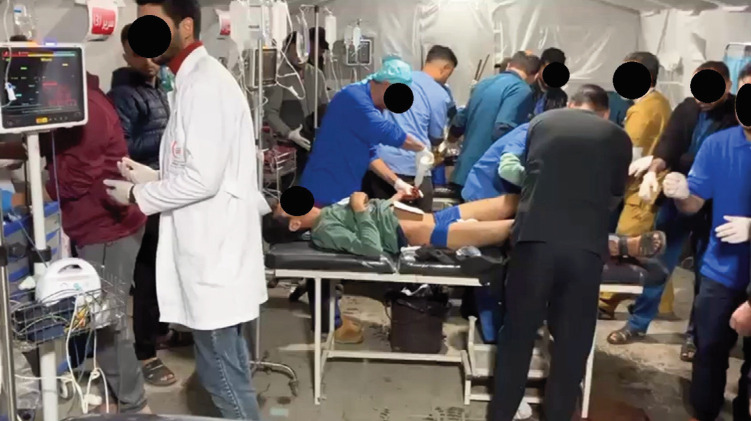
Scene of mass casualty incident in Kuwaiti Specialist Hospital, Rafah. Members of SCT2 providing acute management with local healthcare professionals (photograph courtesy of MERCY Malaysia).

**Fig. 2:**
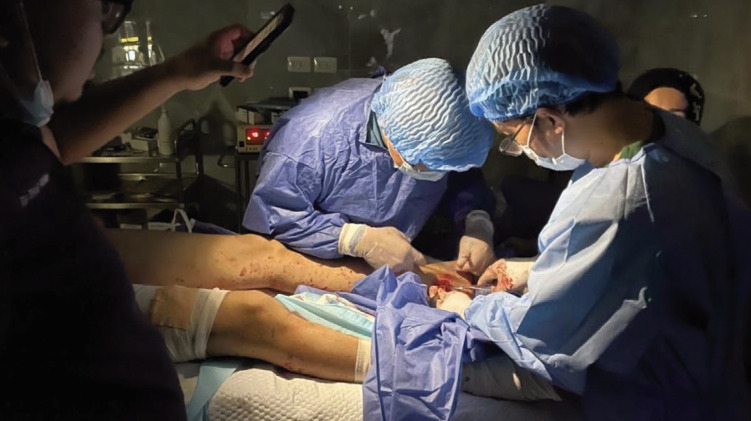
Performing essential treatment prior to ceasefire in the face of inadequate resources including loss of electrical supply, resorting to illumination using mobile phones (photograph courtesy of MERCY Malaysia).

The orthopaedic teams predominantly managed acute musculoskeletal trauma resulting from blast injuries, gunshot wounds, and crush mechanisms from collapsing structures and debris. Their deployment occurred amid ongoing hostilities, with frequent attacks on nearby areas and active drone surveillance. Mass Casualty Incidents (MCI) were common and placed extraordinary strain on limited resources (Fig. 1). Often, only fundamental interventions could be performed due to shortages of surgical consumables, implants, sterilisation equipment, and anaesthetic agents. Recurrent power outages further complicated operative and post-operative care, markedly increasing wound infection rates and impairing wound healing (Fig. 2).

**Fig. 3:**
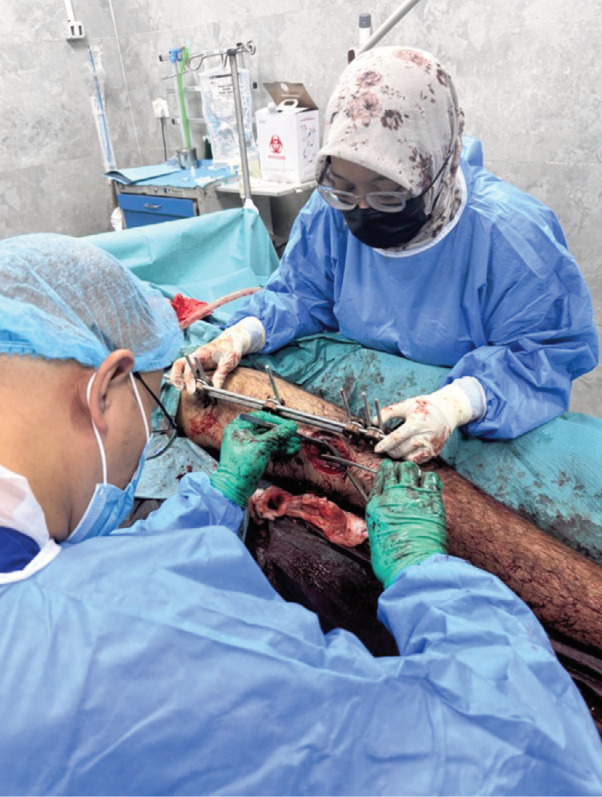
Attending to surgical wound infection and chronic osteomyelitis post-ceasefire consequent to injury severity, sterility restrictions and inadequate supplies (photograph courtesy of MERCY Malaysia).

MM resumed SCT operations shortly before the 10 October 2025 ceasefire, this time entering through the Karim Shalom crossing from Jordan. Since then, multiple teams have been deployed, with rotations planned throughout the remainder of 2025. Injury patterns have shifted from predominantly acute trauma to cases requiring complex salvage and reconstructive procedures. Persistent infection remains a major challenge, driven by the severity of initial open injuries, delayed presentations, and the persistent difficulty in obtaining antibiotics, external fixators, sterile dressings, and other essential supplies (Fig. 3). These materials are frequently restricted as “dual-use” items which required prior authorisation and approval, further impeding care.

Compounding these challenges is the drastic reduction in the number of senior orthopaedic surgeons in Gaza, many of whom have been killed, arrested, or forced to migrate^3^. This has left a significant experience gap among remaining local surgeons. A core focus of the MM SCT teams has therefore been the transfer of expertise—providing supervision, surgical guidance, and technical demonstrations aimed at strengthening local surgical capacity under austere conditions. It is hoped that this knowledge transfer will help sustain competent orthopaedic services should MM teams be withdrawn for any reason.

MM remains committed to delivering essential surgical and medical support in Gaza. We hope more healthcare professionals will volunteer for upcoming missions, and that broader institutional and public support will continue for those undertaking this vital humanitarian work^4^.
